# Application and Preliminary Outcomes of Remote Diagnosis and Treatment During the COVID-19 Outbreak: Retrospective Cohort Study

**DOI:** 10.2196/19417

**Published:** 2020-07-03

**Authors:** Luwen Liu, Jianqin Gu, Fengmin Shao, Xinliang Liang, Lixia Yue, Qiaomei Cheng, Lianzhong Zhang

**Affiliations:** 1 Zhengzhou University People's Hospital Henan Provincial People's Hospital Zhengzhou China

**Keywords:** coronavirus disease, COVID-19, remote diagnosis and treatment, telemedicine, online outpatient visit, offline drug delivery, pandemic management, China, Henan Province

## Abstract

**Background:**

The coronavirus disease (COVID-19) pandemic, caused by the novel severe acute respiratory syndrome coronavirus 2 (SARS-CoV-2), has resulted in the self-quarantine of countless people due to possible infection. This situation makes telemedicine necessary as it can overcome geographical barriers, increase the number of people served, and provide online clinical support for patients. However, the outcomes of telemedicine have not yet been evaluated.

**Objective:**

The aim of our study is to describe the epidemiological features and clinical symptoms of patients receiving remote diagnosis and treatment at the online outpatient clinic of our hospital, as well as to analyze the outcomes and advantages of telemedicine, during the COVID-19 pandemic.

**Methods:**

Data from patients receiving remote diagnosis and treatment via consultation services for COVID-19 concerns at the online outpatient clinic of Henan Provincial People's Hospital from January 24 to February 17, 2020, were collected. A retrospective analysis was performed on epidemiological features, clinical symptoms, and preliminary outcomes.

**Results:**

Online inquiry, consultation, and suggestions were provided for patient concerns related to COVID-19. Our hospital also offered offline noncontact drug delivery services following online ordering and payment. A total of 4589 patients receiving remote diagnosis and treatment were recruited. The daily number of online outpatient visits initially increased and then decreased, reaching its peak on January 28 when the daily number of online outpatient visits totaled 612. Of 4589 patients, 1940 (42.3%) were males and 2649 (57.7%) were females (age range: 78 days to 85 years). Most patients were aged 20-39 years (n=3714, 80.9%) and came from Henan Province (n=3898, 84.9%). The number of patients from other provinces was 691 (15.1%). During the online consultations, patients discussed the following symptoms: fever (n=2383), cough (n=1740), nasal obstruction (n=794), fatigue (n=503), and diarrhea (n=276). A total of 873 orders of noncontact drug delivery following online payment was completed. The daily number of such orders gradually stabilized after the initial, steady increase. For offline drug delivery orders, the median (IQR) was 36 (58). An online satisfaction survey was filled out postconsultation by patients; of the 985 responses received, 98.1% (n=966) of respondents were satisfied with the service they received.

**Conclusions:**

Remote diagnosis and treatment offered via online outpatient consultations effectively reduced the burden on hospitals, prevented overcrowding, reduced the risk of cross-infection, and relieved patients' anxiety during the COVID-19 outbreak. This plays an essential role in pandemic management.

## Introduction

Novel coronavirus (2019-nCoV)–infected pneumonia is caused by the 2019 novel coronavirus [1]. On February 11, 2020, the World Health Organization (WHO) announced the names “coronavirus disease 2019” (COVID-19) for the disease and “severe acute respiratory syndrome coronavirus 2” (SARS-CoV-2) for the virus. According to existing studies, SARS-CoV-2 is closely related to severe acute respiratory syndrome coronavirus (SARS-CoV) and Middle East respiratory syndrome coronavirus (MERS⁃CoV). All three viruses can cause severe symptoms of pneumonia. SARS-CoV-2 is usually found in respiratory secretions and transmitted by droplets (eg, by sneezing and saliva) but can also spread by contact. Its virulence is weaker than SARS⁃CoV, but its transmissibility is higher than SARS⁃CoV. SARS-CoV-2 can spread from person to person, and the general population is susceptible to infection. It is also contagious during the latency period [2-5]. According to the National Health Commission of the People’s Republic of China, it had received reports of 72,528 cumulative confirmed cases of COVID-19 from 31 provinces (autonomous regions and municipalities directly under the central government) and Xinjiang Production and Construction Corps, by 24:00 on February 17, 2020. Other countries, including Thailand, Japan, Korea, and the United States had also reported COVID-19 cases [6-9]. It is difficult to monitor COVID-19 due to its high transmissibility, unclear route of transmission, and atypical symptoms. COVID-19 has posed enormous challenges to China's public health [2].

During the COVID-19 outbreak, nonurgent visits may cause overcrowding in hospitals [10], which not only adds to the burden of hospital staff but also dramatically increases the risk of infection spread. There may be severely adverse consequences for both patients and hospitals, making pandemic control even more difficult. On the other hand, as a major public health event, the COVID-19 pandemic not only endangers people's life and health but also causes psychological stress and anxiety. Active prevention and monitoring of the public’s psychological behavior is an integral part of a reasonable response to public health emergencies. Telemedicine is a health service discipline that combines modern communication, electronic technology, computer networks, and medicine [11]. Telemedicine can overcome geographical barriers, increase the number of people served, and provide online clinical support for patients [12,13]. This novel health service model has great potential for disease prevention and treatment as well as patient nursing during epidemiologic outbreaks [14].

Henan Provincial People's Hospital has opened the COVID-19 online outpatient clinic via the connected smart health service center platform, which provides smart health solutions for patients. The platform has already achieved 5G coverage and combines the internet with medical technology to successfully connect Henan Provincial People's Hospital to online hospitals. Online health services provided to patients include appointment, diagnosis and treatment, payment, quality control, follow-up, hierarchical diagnosis and treatment, emergency rescue, healthy management, public services, logistics, health education, and remote vital signs monitoring. Here, a retrospective analysis was performed to analyze the outcomes and advantages of telemedicine in the context of the COVID-19 outbreak in China.

## Methods

**Figure 1:**
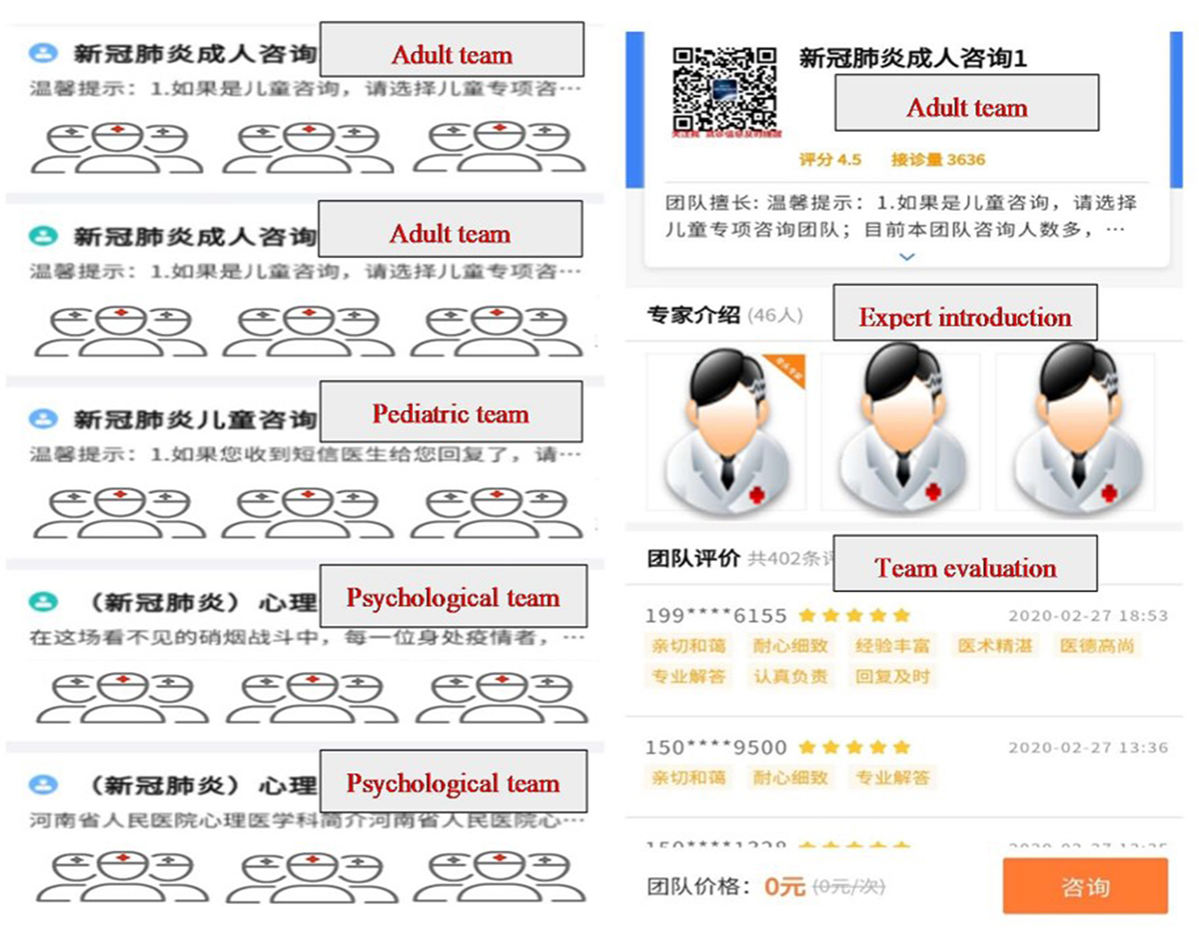
Expert clinician teams.

A retrospective analysis was carried out using data from all patients who received remote diagnosis and treatment via online consultation services provided by our hospital from January 24 to February 17, 2020. During this period, 132 clinicians from Henan Provincial People's Hospital provided online outpatient consultation services to address COVID-19 concerns. These clinicians were from the Department of Infectious Diseases, the Respiratory Department, the Department of Critical Care Medicine, and the Department of Psychology and Psychiatry. They were divided into 5 teams: 2 teams provided consultation services for adult patients (adult team), 1 team for pediatric patients (pediatric team), and 2 teams for patients with psychological problems (psychological team; Figure 1). Online inquiry, consultation, and suggestions were provided to patients for concerns related to COVID-19 and the services were free of charge. Meanwhile, the hospital offered offline noncontact drug delivery services following online ordering and payment.

**Figure 2:**
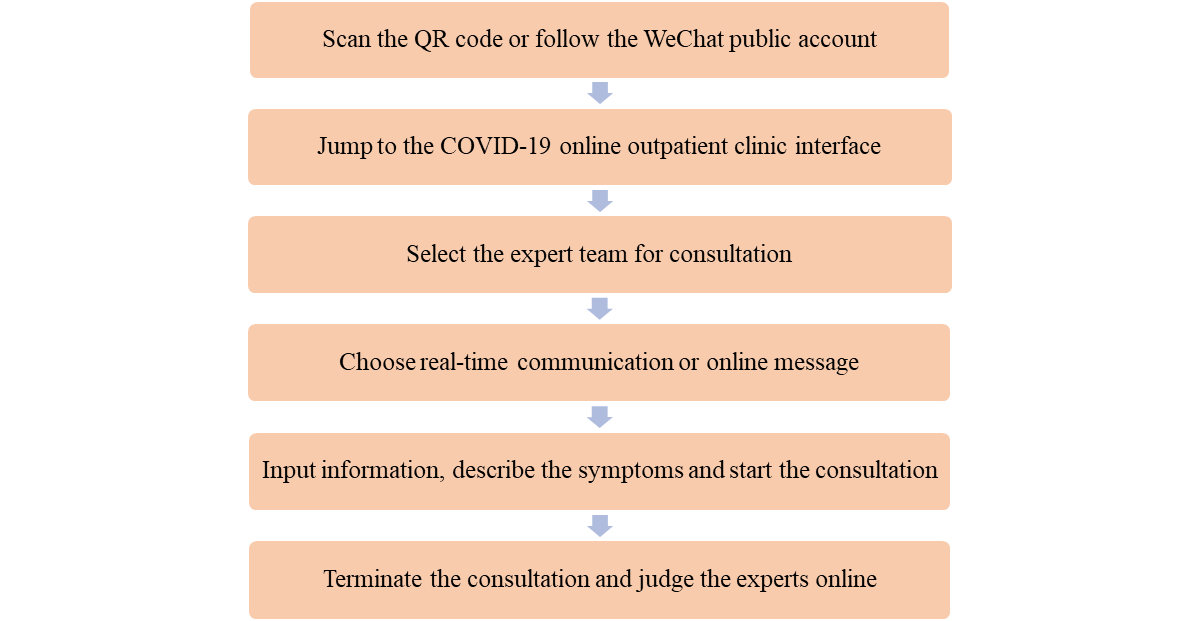
Flow chart of remote diagnosis and treatment via the online outpatient clinic. The platform supports consultations to be conducted under any network conditions. COVID-19: coronavirus disease; QR: Quick Response.

**Figure 3:**
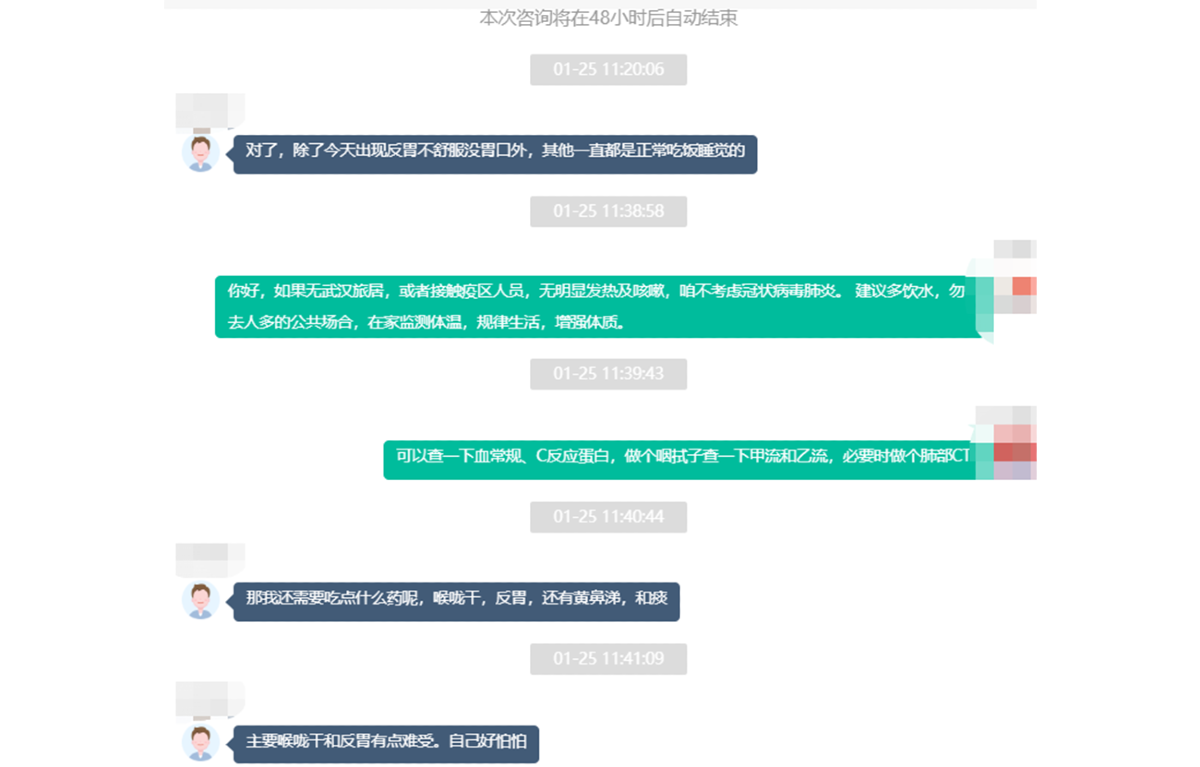
The patient-doctor dialogue interface for online outpatient consultation.

In order to consult with a clinician, patients can scan the official QR (Quick Response) code or follow the WeChat public account HNFYSY1904, which directs them to the expert consultation interface and allows access to the online outpatient consultation. Alternatively, patients can directly search our health service website to access the consultation page. Following the prompts, the patients click on the “Consult” button in the lower right corner, then input personal information and a description of symptoms, and start the consultation (Figure 2). The patients can choose real-time communication or online messaging for inquiry and consultation. The patients can interact with the clinicians online via voice, text, photo, and video (Figure 3).

During telemedicine consultations, we defined mild illness as follows: fever below 38℃ and no history of epidemiological exposure. Patients with a fever greater than 38℃ or with a history of epidemiological exposure were advised go to the hospital immediately. For mildly symptomatic patients and those with symptoms like cough, nasal congestion, fatigue, diarrhea, etc, our procedure was to provide medication guidance, recommend home isolation, and regular temperature monitoring. In cases where the temperature exceeds 38℃ or symptoms worsen, we recommended patients to visit a hospital for treatment.

At the end of the remote consultation, the platform will automatically open an electronic questionnaire, which the patients have the option to fill. The questionnaire includes star ratings and open answers. A minimum of 1 star represents high dissatisfaction, and a maximum of 5 stars represents high satisfaction. Four stars and above represent satisfaction. Following this, patients can input their own opinions and suggestions.

Since the online outpatient consultation service first began on January 24, 2020, online remote diagnosis and treatment and offline drug delivery services have been provided nonstop every day from 8:00 to 22:00. Patients’ personal information is stored on the connected smart health service center’s cloud-based platform, which ensures data safety. All statistical analyses were performed using SPSS 18.0 software (IMB Corporation). Categorical variables were expressed as absolute and relative frequencies and percentages.

The study protocol was approved by the ethics committee of Henan Provincial People's Hospital (Zhengzhou, China).

## Results

In total, 4589 patients received remote diagnosis and treatment via the COVID-19 online outpatient clinic. Among them, there were 1940 (42.3%) males and 2649 (57.7%) females. The youngest patient was aged 78 days, and the eldest was 85 years old. In terms of age, 320 patients were aged <20 years, 2007 patients were aged 20-29 years, and 1707 were aged 30-39 years; the latter 2 groups accounted for 80.9% (n=3714) of the sample. Additionally, 315 patients were aged 50-59 years, and 50 patients were ≥60 years. Most patients (n=3898, 84.9%) came from Henan Province; 691 (15.1%) patients came from other provinces. During the epidemic, all patients with suspected COVID-19 received by a hospital were referred to a fever clinic. A total of 4561 patients were treated in the fever clinic of our hospital. Among them, there were 2375 (52.1%) male patients and 2186 (47.9%) female patients.

**Figure 4:**
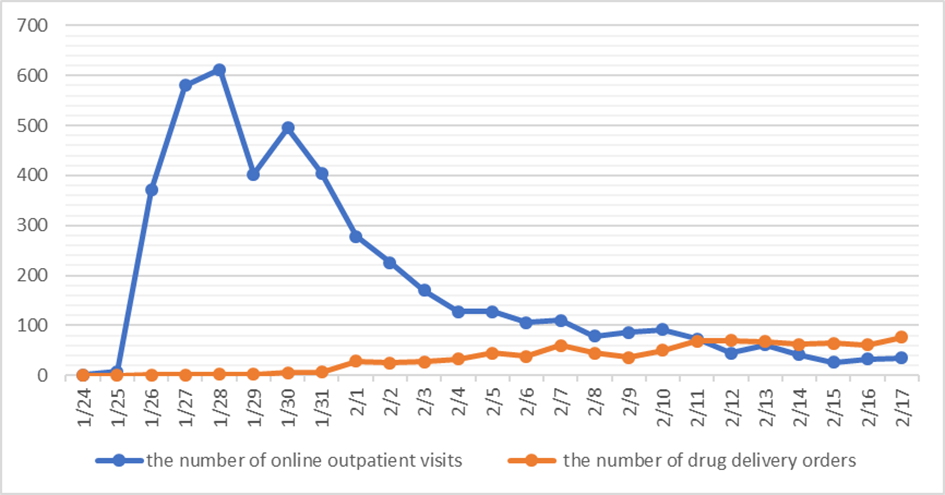
The daily number of online outpatient visits and offline noncontact drug delivery orders.

From January 24 to February 17, 2020, the daily number of online outpatient visits first increased and then decreased, reaching its peak on January 28 when the daily number of online outpatient visits reached 612. Following this, the second peak occurred on January 30 when visits totaled 495. This number gradually decreased afterward. Between February 15-17, the daily number of online outpatient visits remained at 25-35, with minimal change. The number of offline noncontact drug delivery orders following online payment first increased and then stabilized. The total number of offline noncontact drug delivery orders was 873. As shown in Figure 4, after February 12, the daily number of noncontact drug delivery orders exceeded that of online outpatient visits before gradually stabilizing.

By February 17, the 5 expert teams had provided remote consultation services for 4589 patients (Table 1). In total, the 2 adult teams provided consultations to 4399 adult patients, accounting for 95.86% of the sample; the pediatric team provided consultations to 80 (1.74%) pediatric patients; and the 2 psychological teams provided psychological counseling for 110 (2.40%) patients (Table 1). The content of the consultations was classified according to the symptoms concerned (one patient might have one or more symptoms), including fever, headache, cough, fatigue, nasal obstruction, sore throat, nasal obstruction, and diarrhea. The most common symptom that patients initiated a consultation for was fever (n=2383 patients), followed by cough (n=1740), nasal obstruction (n=794 patients), fatigue (n=503), and diarrhea (n=276). Among the 110 patients who received psychological counseling, 7 patients reported psychological stress and anxiety due to their history of epidemiological exposure. The remaining 103 patients had no history of epidemiological exposure.

**Table 1:** Total number of online outpatient visits handled by clinicians for concerns related to coronavirus disease (COVID-19) (N=4589).

Consultation type	Clinicians, n	Online outpatient visits, n (%)
Consultation for adult patients	77	4399 (95.86)
Consultation for pediatric patients	28	80 (1.74)
Psychological counseling	27	110 (2.40)

Among the 4589 patients who received remote consultation services, 310 patients were advised to go to the hospital for further examination due to fever greater than 38℃, and 301 patients were advised to visit a hospital for further examination because of a history of epidemiological exposure and other symptoms. In total, 985 patients responded to the satisfaction questionnaire, of whom 98.1% (n=966) were satisfied with the service (rated as 4 stars or above). They praised the doctors for their rich experience, professional answers, dedication to their work, timely response, and patience. In the 110 cases of psychological counseling, 47 cases provided feedback, and the proportion of satisfaction ratings greater than 4 stars was 100%.

## Discussion

### Principal Findings

The COVID-19 outbreak occurred suddenly and spread rapidly and extensively. The disease is characterized by a long latency period and is even contagious during latency. The WHO attributed a “very high” risk level to COVID-19 on January 28, 2020. Cutting off sources of infection and reducing cross-infection through quarantine, disinfection, and personal protection are the top priorities of countries around the world [15]. Markwell et al [16] showed that the risk of cross-infection in hospitals could be decreased by reducing the visitor flow rate and chances of virus exposure in hospitals. Such countermeasures have already been widely adopted during the outbreak of many other contagious diseases [16-18]. Our hospital launched the online outpatient clinic services to address COVID-19 concerns via telemedicine. This online outpatient service platform can provide patients prehospital guidance, support online payment and offline drug distribution, reduce the possibility of cross-infection caused by nonurgent visits to offline clinics, and relieve the patient's mental health problems. It is of great significance for the prevention and treatment of pandemics.

In the present study, 80.9% of the online outpatient visits were from patients in the 20-39–year age group (n=3714), whereas patients aged ≥60 years made up only 1.1% (n=50) of the sample. The following reason is proposed to explain this phenomenon: young people are better adapted to use the internet. In the information era, young people prefer to use the internet to solve less complex health problems [14]. According to the latest Chinese statistical report on internet development released by the China Internet Network Information Center in 2019 [19], combined with the age distribution of the Chinese population, internet users aged 10-39 years account for 65.1% of the total number of internet users. Within this group, internet users aged 20-29 years account for the highest proportion (24.6%), and about 91.3% of the total population of the same age group; internet users aged 30-39 years account for 23.7%, and about 94.1% of the total population of the same age group; internet users aged ≥60 years account for 6.9%, and about 23.2% of the total population of the same age group. These data suggest that the internet is indeed more widely used by young people, which is an important reason for the increased use of telemedicine in this cohort.

During the pandemic, the number of online outpatient remote consultations and offline fever clinic visits was 4589 and 4561, respectively. The number of remote consultations via the online clinic was comparable to the total number of cases that the fever clinic handled in the same period. Although these data cannot directly show that telemedicine has reduced the total number of cases in hospitals, it is certain that telemedicine has reduced the burden on hospitals to some extent. Fewer outpatient visits allow hospitals to avoid overcrowding while reducing the risk of hospital cross-infection.

As shown in Figure 4, the number of patients who received remote consultation regarding COVID-19 concerns first increased and then decreased. After the first peak on January 28, a minor peak occurred on January 30. Later, the daily number of online outpatient visits gradually decreased. In the meantime, the daily number of offline noncontact drug delivery orders following online payment steadily increased. After February 12, the daily number of noncontact drug delivery orders exceeded that of online outpatient visits and gradually stabilized. According to National Health Commission statistics [20], Henan Province had a cumulation of 32 confirmed cases by January 24. At this time, the online outpatient consultation service had just been launched, and few people knew about the service. The daily number of online outpatient visits was only 1, and offline drug delivery was not yet available. By January 28, Henan Province had 206 confirmed cases [20]. This was also the day when the daily number of online outpatient visits reached a peak (n=612). On the contrary, the daily number of offline drug delivery remained low, since many people were afraid of personal contact. The daily number of newly confirmed cases increased considerably in Henan Province in the following days [20], and the general public began to gain more knowledge about the COVID-19 outbreak. Those with more severe symptoms would go to a hospital for further examination, resulting in a mild increase in the daily number of offline drug delivery orders. From February 13-17, the daily number of newly confirmed cases was not above 20 in Henan Province [20]. Most people had already been practicing self-quarantine for over 14 days. The daily number of online outpatient visits fluctuated at a low level, and the daily number of offline noncontact drug delivery orders stabilized.

According to the *Diagnosis and Treatment Protocol for Novel Coronavirus-Infected Pneumonia (Version 6)* released by the National Health Commission on February 19, 2020, and the latest research findings, fever, fatigue, and dry cough are the primary symptoms of COVID-19 [21]. Nasal obstruction, runny nose, and other upper respiratory symptoms are rare [22]. Gastrointestinal symptoms are also uncommon [23]. This study demonstrates that, among all remote consultations, 2383 cases exhibited fever symptoms and 1740 cases had cough symptoms, which is in agreement with the fact that fever and cough are the primary clinical symptoms of COVID-19. Nasal obstruction, a rare symptom of COVID-19, was the third frequent symptom that patients consulted for, and fatigue ranked fourth in the number of consultations; this is inconsistent with reported primary symptoms of COVID-19. The fifth most common symptom was diarrhea, which is also uncommon among COVID-19 patients. It is assumed that the symptoms consulted for by patients are more common symptoms of infection, and there is no precise connection with the main symptoms of COVID-19.

Previous research has demonstrated that over 58% of the respondents have psychological health problems amidst public health emergencies and have a strong need for psychological intervention [24-26]. As a result, some people may suffer from acute stress disorder, posttraumatic stress disorder, depression, other psychological disorders, or even commit suicide [27]. Most patients with psychological disorders may not be willing to go to the hospital for consultation due to the risks of cross-infection [28]. Telemedicine provides a way for patients to alleviate their psychological problems. In total, 110 patients received professional psychological counseling through our hospital’s online outpatient consultation service. The clinicians taught them simple methods to relieve anxiety, including relaxation training through online tutorials, deep breathing exercises, soothing music, etc. In total, 47 patients provided feedback on the psychological services they received. All 47 patients provided ratings greater than 4 stars and reported that after the remote consultation, they had eliminated their doubts, reduced their anxiety, and were satisfied with the consulting doctor. The data suggest that remote counseling can be helpful in relieving anxiety.

### Limitations

The biggest limitation of the present study is the absence of follow-up due to the already heavy burden of medical staff. Therefore, there is no information about follow-up, which is not conducive for further studies. Secondly, because the satisfaction questionnaire was filled out voluntarily by patients rather than obtained by random sampling, the results may be biased.

### Conclusion

During the COVID-19 outbreak, telemedicine can quickly establish online services within a short timeframe. This can reduce the burden of hospital personnel and the risk of cross-infection caused by offline treatment visits, as well as relieve the psychological burden and anxiety of patients to a certain extent. Telemedicine, therefore, plays a crucial role in the prevention and management of the pandemic.

## Abbreviations

2019-nCoV: novel coronavirus
COVID-19: coronavirus disease
MERS⁃CoV: Middle East respiratory syndrome coronavirus
QR: Quick Response
SARS-CoV: severe acute respiratory syndrome coronavirus
SARS-CoV-2: severe acute respiratory syndrome coronavirus 2
WHO: World Health Organization
